# Restoration of Proresolution Pathway with Exogenous Resolvin D1 Prevents Sevoflurane-Induced Cognitive Decline by Attenuating Neuroinflammation in the Hippocampus in Rats with Type 2 Diabetes Mellitus

**DOI:** 10.3389/fphar.2021.720249

**Published:** 2021-07-23

**Authors:** Qingmei Sun, Hongdan Yan, Falong Chen, Fen Jiang, Wenjuan Chen, Dongliang Li, Yongmin Guo

**Affiliations:** Department of Anesthesiology, Qilu Hospital, Shandong University, Jinan, China

**Keywords:** sevoflurane, type 2 diabetes mellitus, resolvins, cognitive decline, inflammation

## Abstract

Sevoflurane (SEV), a commonly used volatile anesthetic, has been shown to cause cognitive decline in diabetic rats by aggregating neuroinflammation in the hippocampus, but the underlying mechanisms are unknown. Recent evidence suggests that neuroinflammation could be a consequence of failure to resolve inflammation by specialized pro-resolving lipid mediators including resolvin D1 (RvD1). Here we first examined whether type 2 diabetes mellitus (DM) alters RvD1 proresolution pathway. Diabetic Goto-Kakizaki (GK) rats and non-diabetic Wistar rats received control or 2.6% SEV exposure for 4 h. Seven days after exposure, GK control rats, compared with Wistar control rats, had significantly lower RvD1 levels in plasma and CSF and decreased RvD1 receptor FPR2 expression in the hippocampus. SEV increased RvD1 levels in plasma and CSF and FPR2 expression in the hippocampus in Wistar rats but not in GK rats. We next examined whether RvD1 treatment of GK rats can prevent SEV-induced neuroinflammation and cognitive decline. GK rats received control, SEV or SEV and once-daily treatment with exogenous RvD1 (0.2 ug/kg, ip) for 7 days. RvD1 administration markedly increased RvD1 levels in plasma and CSF and FPR2 expression in the hippocampus in GK rats received SEV. Compared with GK control rats, GK rats received SEV exhibited shorter freezing times in trace fear conditioning task, which was accompanied by increased microglia activity and pro-inflammatory cytokine expression in the hippocampus. RvD1 administration attenuated SEV-induced increases in microglia activity and pro-inflammatory cytokine expression in the hippocampus, preventing cognitive decline in GK rats. Notably, neither SEV nor RvD1 altered metabolic parameters in GK rats. The results suggest that RvD1 proresolution pathway is impaired in the brain of diabetic GK rats. which may enhance the susceptibility to SEV, contributing to neuroinflammation and cognitive decline. Restoration of RvD1 proresolution pathway in diabetic GK rats with exogenous RvD1 can prevent SEV-induced cognitive decline by attenuating neuroinflammation in the hippocampus.

## Introduction

Post-operative cognitive dysfunction (POCD) is characterized by a decline in cognitive performance following anesthesia and surgery when compared to preoperative cognitive status (Skvarc et al., 2018). POCD may last from days to months, and in some rare cases, even up to 1–2 years after surgery (Tsai et al., 2010). The incidence of POCD has been observed to be approximately 10% of all surgical patients and 40% of elderly patients at the point of discharge and the long-term consequences of this are marked by a significantly higher mortality rate than age and sex-matched controls without POCD (Skvarc et al., 2018). Although the precise mechanism underlying POCD remains elusive, preclinical and clinical studies have shown that cognitive impairment can be induced by general anesthetics (Jevtovic-Todorovic et al., 2013; Li et al., 2017; Zhang et al., 2019; Li and Zhang, 2021). Of note, cognitive impairment caused by general anesthetics is dependent on the choice of anesthesia agent, doses of the drug, time or duration of anesthesia administration and patient age (Shen et al., 2013; Callaway et al., 2015). Moreover, a pre-existing medical condition such as type 2 diabetes mellitus (DM) or chronic intermittent hypoxia may enhance the vulnerability to the development of POCD following anesthesia (Feng et al., 2013; Yang et al., 2014; Yue et al., 2015; Li et al., 2017; Zhang et al., 2019).

Accumulating evidence reveals that neuroinflammation in the hippocampus, a brain structure that plays an important role in learning and memory, is involved in the pathogenesis of POCD (Carlini et al., 2011; Liu and Yin, 2018; Skvarc et al., 2018; Li and Zhang, 2021). Microglial priming is a key mediator of neuroinflammation, primarily in the hippocampus, following a variety of peripheral insults, including anesthetic exposure (Barrientos et al., 2015; Norden et al., 2015). Neuroinflammation can lead to deteriorated cellular and molecular processes important for forming memories, which, in turn, induces precipitous short-term or long-term memory deficits (Barrientos et al., 2006; Spencer et al., 2017; Muscat et al., 2021). Interventions to inhibit microglia activity and neuroinflammation in the brain have been shown to improve cognitive dysfunction in many neurodegenerative diseases (Wadhwa et al., 2017; Zhang et al., 2017).

Sevoflurane (SEV), a volatile anesthetic, has been widely used to induce and maintain general anesthesia in both adult and pediatric patients during surgery due to its favorable clinical characteristics such as rapid pharmacokinetics and lack of airway irritability (Brioni et al., 2017). Preclinical studies have reported that a moderate duration of SEV (2–3% for 2 or 4 h) does not cause cognitive impairment in adult or aged animals (Callaway et al., 2012; Shen et al., 2013), however, it induces persistent cognitive decline in type 2 diabetic animals by promoting microglia activity and aggravating neuroinflammation in the hippocampus (Li et al., 2017).

Recent studies suggest that neuroinflammation could be a consequence of failure to resolve inflammation and to restore tissue homeostasis (Serhan, 2014; Chiurchiu et al., 2018; Trojan et al., 2020). The resolution of inflammation is mediated by specialized pro-resolving lipid mediators (SPMs) including D-series resolvins (RvDs), which are endogenous lipid mediators derived from n-3 polyunsaturated fatty acids with both anti-inflammatory and pro-resolutive activities (Serhan, 2014; Chiurchiu et al., 2018; Trojan et al., 2020; Tiberi and Chiurchiu, 2021). Among the resolvins, resolvin D1 (RvD1) is of particular interest in the resolution of inflammation because it actively turns off the inflammatory response (Fredman and Serhan, 2011). Defects in the RvD1 proresolution pathway have been shown to contribute to the progression of chronic inflammation in the peripheral tissues following insults (Chiurchiu et al., 2019). In addition, RvD1 treatment promotes resolution of inflammation in the microglia cells in response to lipopolysaccharide (LPS) challenge (Rey et al., 2016; Tiberi and Chiurchiu, 2021).

In this study, we examined whether type 2 DM alters RvD1 proresolution pathway and if so, whether RvD1 treatment can prevent SEV-induced neuroinflammation and cognitive decline in a rat model with type 2 DM.

## Methods

### Animals

Male diabetic Goto-Kakizaki (GK) rats, a genetic non-obese model of type 2 DM, and age-matched male non-diabetic Wistar rats (30–32 weeks old) were obtained from SLAC Laboratory Animal, Co., Ltd. (Shanghai, China). All Animals were maintained under constant temperature (21°C) and were given free access to tap water and standard rat chow. All animal procedures were conducted according to the guidelines of the Animal Care and Use Committee at Shandong University and were approved by the Animal Care and Use Committee at Shandong University.

### Experimental Design

Protocol I: To examine whether type 2 DM altered RvD1 proresolution pathway, GK rats and Wistar rats were assigned to the following groups (n = 8 rats per group): 1) GK control rats (GK + CON), 2) GK rats that received SEV exposure (GK + SEV), 3) Wistar control rats (Wistar + CON), and 4) Wistar rats that received SEV exposure (Wistar + SEV). For animals that were assigned to SEV exposure, 2.6% SEV was given by a humidified 30% O2 carrier gas from a calibrated vaporizer for 4 h, as described previously (Li et al., 2017). Animals that were assigned to control groups were placed in the same chamber except that SEV was not provided. The concentrations of SEV, O2 and CO2 in the chamber were continuously monitored using a gas analyzer (Datex Ohmeda, Mississauga, ON, Canada). Seven days after SEV exposure, these animals were sacrificed to collect blood, cerebrospinal fluid (CSF) and brain samples for biochemical and molecular studies.

Protocol II: To examine whether RvD1 treatment of diabetic rats could prevent SEV-induced neuroinflammation and cognitive decline, GK rats were assigned to the following groups (n = 16 rats per group): 1) GK control rats (GK + CON), 2) GK rats that received SEV exposure (GK + SEV), and 3) GK rats that received SEV exposure and once-daily intraperitoneal (ip) injection of RvD1 (0.2 ug/kg), starting 30 min prior to SEV exposure for the first injection (GK + SEV + RvD1). The SEV exposure was performed as described in protocol I. The dose of RvD1 was based on a previous study in which this dose could increase plasma and brain RvD1 levels and reduce microglia-mediated neuroinflammation in the brain in rats (Krashia et al., 2019). Seven days after SEV exposure, trace-fear conditioning (TFC) and open field test were performed in some animals (n = 8 for each group) to assess effects of RvD1 treatment on cognitive function and locomotor activity. These animals were then sacrificed to obtain blood, CSF and brain samples for biochemical and molecular studies. The rest of animals from each group (n = 8 rats per group) were transcardially perfused with 4% paraformaldehyde for immunofluorescence study.

### Trace-Fear Conditioning Task

The TFC task was used to examine the hippocampal-dependent learning and memory alterations as previously described (Li et al., 2017). The TFC was performed in a fear conditioning apparatus chamber (Med Associates, Inc.) consists of a grid floor bottom which was used to deliver a mild foot shock. The testing chamber was housed in a room with overhead fluorescent light and a ventilation fan providing background noise (65 db). On the training day, rats were transported to the chamber and allowed to explore the chamber for 3 min. After which, three consecutive pairs of tone (80 dB, 5 kHz, 20 s) and foot shock (0.8 mAmp, 2 s) with an empty trace interval of 20 s and a break between each pair of 3 min were administered to the animals.

SEV exposure was carried out within 30 min after TFC training. Memory of the learned fear was examined 1 week later by returning the animals into the original chamber in which they were trained but without any tone or shock. Behavior for each animal was recorded and scored every 5 s during the 5 min observation period. A percentage was calculated using formula 100 × f/n, where f is the number of freezing events (absence of all movement except for respiration) per animal and *n* is the total number of observations per animal.

### Open-Field Test

Open Field test was used to access general locomotor activity as previously described (Li et al., 2017). Briefly, animals were brought into the testing room and allowed to acclimate to the testing room for a minimum of 30 min prior to starting the test. The animals were then placed in an open field apparatus in which the floor was subdivided into 25 blocks with thin white stripes. Each animal was allowed to explore the apparatus for 5 min while animal activity was recorded by a video camera and the automated tracking system. The apparatus was cleaned with 75% alcohol before testing each animal. The number of line crossing and the frequency of rearing performed in a 5-min period was scored.

### Biochemical Assay

Blood was collected and immediately centrifuged at 2,500 g for 15 min at 4°C to isolate plasma. The levels of RvD1 and RvD2 in plasma and CSF and the levels of insulin in plasma were measured using commercially available enzyme-linked immunosorbent assay (ELISA) kits (MyBioSource, Inc. San Diego, CA, United States and Invitrogen, Camarillo, CA, United States, respectively) according to manufacturer’s instructions. The glucose levels in plasma were measured using a glucose analyzer (Prestige Smart System).

### Immunoblotting

The hippocampus was homogenized in ice-cold lysis buffer containing protease inhibitor cocktails. Equal protein amounts were electrophoresed on a 10% SDS-PAGE gel and subsequently transferred to PVDF membranes. The membrane was blocked with 5% nonfat milk for 1 h at 24°C, followed by incubation overnight at 4°C with primary antibodies to RvD 1 receptor FPR2 (Thermo Fisher Scientific, Rockford, IL, United States), proinflammatory cytokines tumor necrosis factor (TNF)-α, interleukin (IL)-1β and IL-6 (Abcam, Cambridge, MA, United States), and *β*-actin (Santa Cruz Biotechnology, CA, United States). Blots were then incubated with HRP-conjugated second antibodies (Santa Cruz Biotechnology, CA, United States) for 1 h. The immunoreactive bands were visualized with ECL Plus reagents (Amersham Biosciences Inc. Piscataway, NJ) and developed on a film. Band density measurements were made using ImageJ software e (NIH, Bethesda, MD, United States).

### Immunofluorescence Study

Immunofluorescence staining for microglia was conducted as described before (Li et al., 2017). Briefly, rats were perfused transcardially with ice-cold salt solution followed by 4% paraformaldehyde (PFA) in PBS. Brains were harvested and postfixed in 4% PFA overnight and then with 30% sucrose for 24 h. Brains were freeze-mounted in optimal cutting temperature (OCT) embedding medium and coronal hippocampal sections were cut sequentially at 20-μm using a cryostat. Sections were permeabilized in PBS with 0.5% Triton X-100, blocked with 5% bovine serum albumin in PBS for 60 min at room temperature, and incubated with anti-CD11b primary antibody (clone OX-42, Chemicon, Temecula, CA, United States) at 4°C overnight. Subsequently, the sections were incubated in an anti-mouse secondary antibody (Alex Fluor 488, Invitrogen, Carlsbad, CA, United States) for 1 h in dark at room temperature. After washing in PBS, sections were mounted on glass slides and coverslipped with fluorescence mounting medium. A confocal microscope (Zeiss LSM 510; Carl Zeiss, Thornwood, NY, United States) was used to detect immunofluorescence of microglia. The number of activated microglia, which was defined by stronger CD11b staining, the presence of a clearly enlarged Soma and marked changes in the appearance of the processes (Rana et al., 2010), were counted and expressed as a percentage of the total number of microglia.

### Statistical Analyses

Statistical analysis was performed using GraphPad Prism 6.0 (GraphPad Software, San Diego, CA, United States). All data were expressed as means ± SEM. Statistical significance was determined using one-way or two-way ANOVA followed by a Bonferroni post hoc test. Values were considered statistically significant when *p* < 0.05.

## Results

### The Resolvin D1 Proresolution Pathway is Impaired in Diabetic Goto-Kakizaki Rats in Response to Sevoflurane Exposure

At the end of the study protocol, body weight and plasma insulin levels were significantly lower, but plasma glucose concentrations were higher in GK + CON rats compared with Wistar + CON rats (Table 1). These data indicate the development of type 2 DM in GK rats during the experimental period. SEV exposure did not change any of these metabolic parameters in Wistar rats or GK rats.

**TABLE 1:** Metabolic parameters in Wistar and GK rats.

Metabolic variables	Wistar + CON	Wistar + SEV	GK + CON	GK + SEV
Body weight (g)	427 ± 5	428 ± 7	375 ± 8^a^	382 ± 7^a^
Glucose (mg/dl)	110 ± 5	112 ± 4	237 ± 8^a^	228 ± 7^a^
Insulin (μg/L)	2.67 ± 0.16	2.78 ± 0.20	1.27 ± 0.15^a^	1.31 ± 0.18^a^

Data are presented as mean ± SE (n = 8 for each group).

a *p* < 0.05 versus Wistar + CON or Wistar + SEV.

**FIGURE 1:**
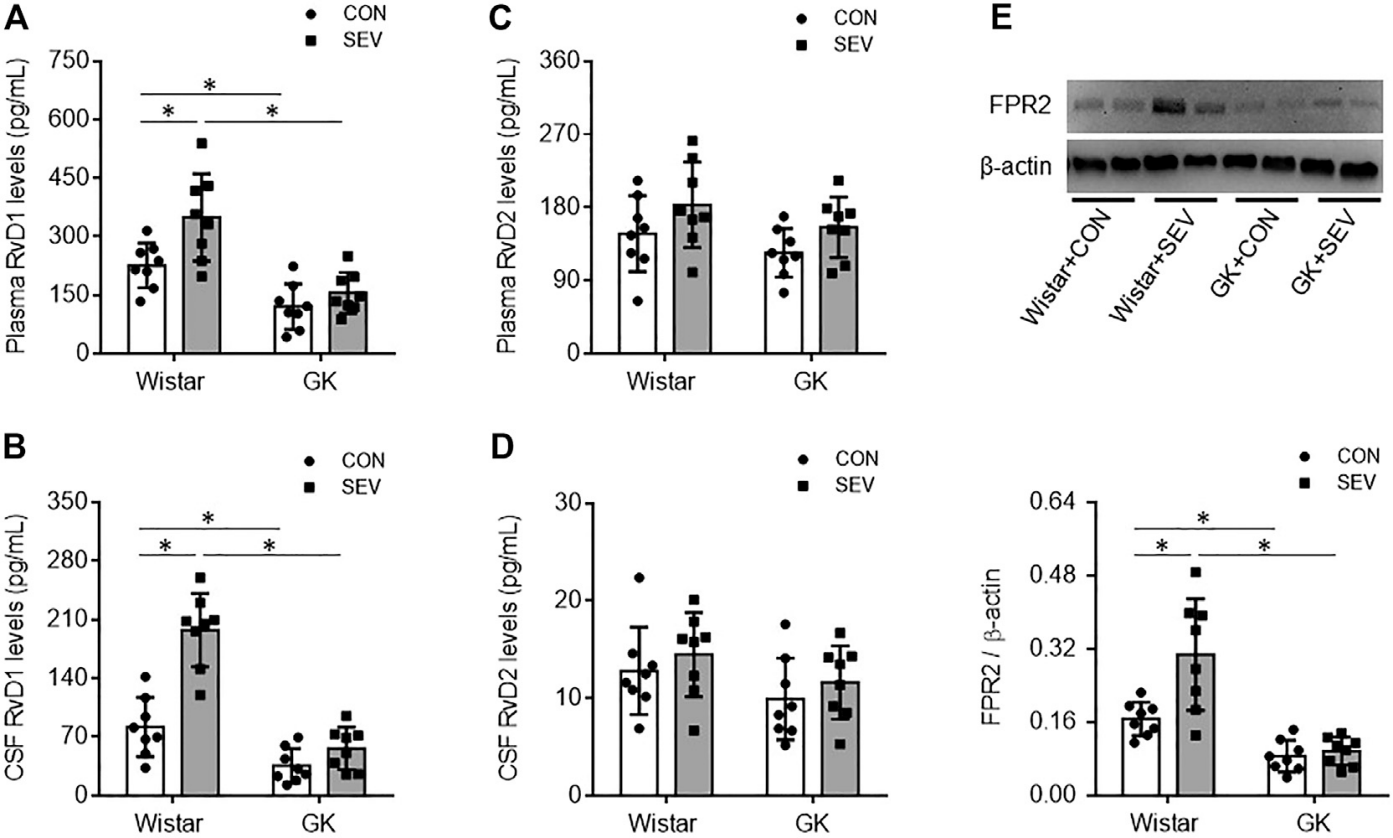
Levels of resolvin D1 (RvD1) and resolvin D2 (RvD2) in plasma **(A and C)** and cerebrospinal fluid (CSF) **(B and D)** and protein expression of their receptor FPR2 in the hippocampus **(E)** in non-diabetic Wistar rats and type 2 diabetic Goto–Kakizaki (GK) rats after control (CON) or sevoflurane (SEV) exposure. Data are presented as mean ± SE (n = 8 for each group). *p < 0.05.

Our previous study has shown that SEV exposure induces neuroinflammation in the hippocampus in diabetic GK rats but not in non-diabetic Wistar rats (Li et al., 2017). To examine whether RvD1 proresolution pathway might be altered in diabetic GK rats in response to SEV exposure, we measured the levels of RvD1 and RvD2 in plasma and CSF and expression of their receptor FPR2 in the hippocampus in GK rats and Wistar rats. Although both RvD1 and RvD2 were detected in plasma and CSF, the levels of RvD1 in both plasma (Figure 1A) and CSF (Figure 1B) in GK + CON rats were significantly lower than in Wistar + CON rats. However, the levels of RvD2 in plasma (Figure 1C) or CSF (Figure 1D) were comparable between the two CON groups. Western blot analysis revealed that protein expression of FPR2 was downregulated in the hippocampus in GK + CON rats compared with Wistar + CON rats. Notably, SEV exposure markedly increased levels of RvD1 in plasma and CSF and upregulated protein expression of FPR2 in the hippocampus in Wistar rats but not in GK rats. No alterations were observed in levels of RvD2 in GK rats or Wistar rats after SEV exposure.

### Effects of Exogenous Resolvin D1 Treatment on Sevoflurane-Induced Neuroinflammation in Diabetic Goto-Kakizaki Rats

**FIGURE 2:**
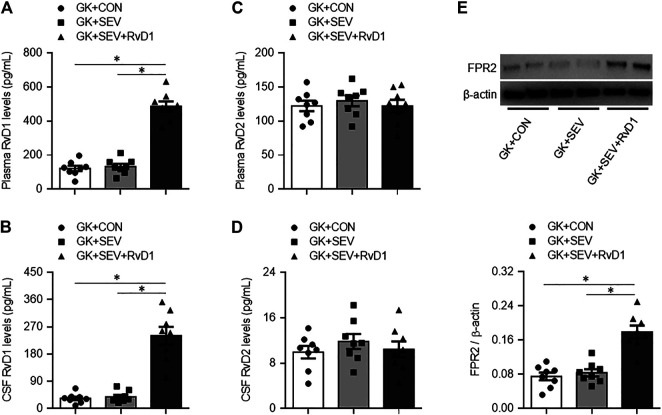
Levels of resolvin D1 (RvD1) and resolvin D2 (RvD2) in plasma **(A and C)** and cerebrospinal fluid (CSF) **(B and D)** and protein expression of their receptor FPR2 in the hippocampus **(E)** in type 2 diabetic Goto-Kakizaki (GK) rats after control (CON) or sevoflurane (SEV) exposure and in exogenous RvD1-treated GK rats after SEV exposure. Data are presented as mean ± SE (n = 8 for each group). *p < 0.05.

Given the defects in endogenous RvD1 proresolution pathway in diabetic GK rats, we next tested the pharmacological action of exogenous RvD1 in neuroinflammation induced by SEV exposure. Exogenous RvD1 administration for 7 days had no effects on body weight, plasma insulin levels or plasma glucose concentrations in GK rats received SEV exposure (Table 2). GK + SEV rats treated with exogenous RvD1 displayed significantly higher levels of RvD1 in plasma (Figure 2A) and CSF (Figure 2B) and augmented protein expression of FPR2 in the hippocampus (Figure 2E), compared with GK + CON rats or GK + SEV rats. There were no differences in levels of RvD2 in plasma (Figure 2C) and CSF (Figure 2D) among three experimental groups.

**TABLE 2:** Metabolic parameters in GK rats.

Metabolic variables	GK + CON	GK + SEV	GK + SEV + RvD1
Body weight (g)	358 ± 11	360 ± 8	373 ± 9
Glucose (mg/dl)	241 ± 10	239 ± 9	231 ± 7
Insulin (μg/L)	1.29 ± 0.12	1.31 ± 0.19	1.26 ± 0.17

Data are presented as mean ± SE (n = 8 for each group).

**FIGURE 3:**
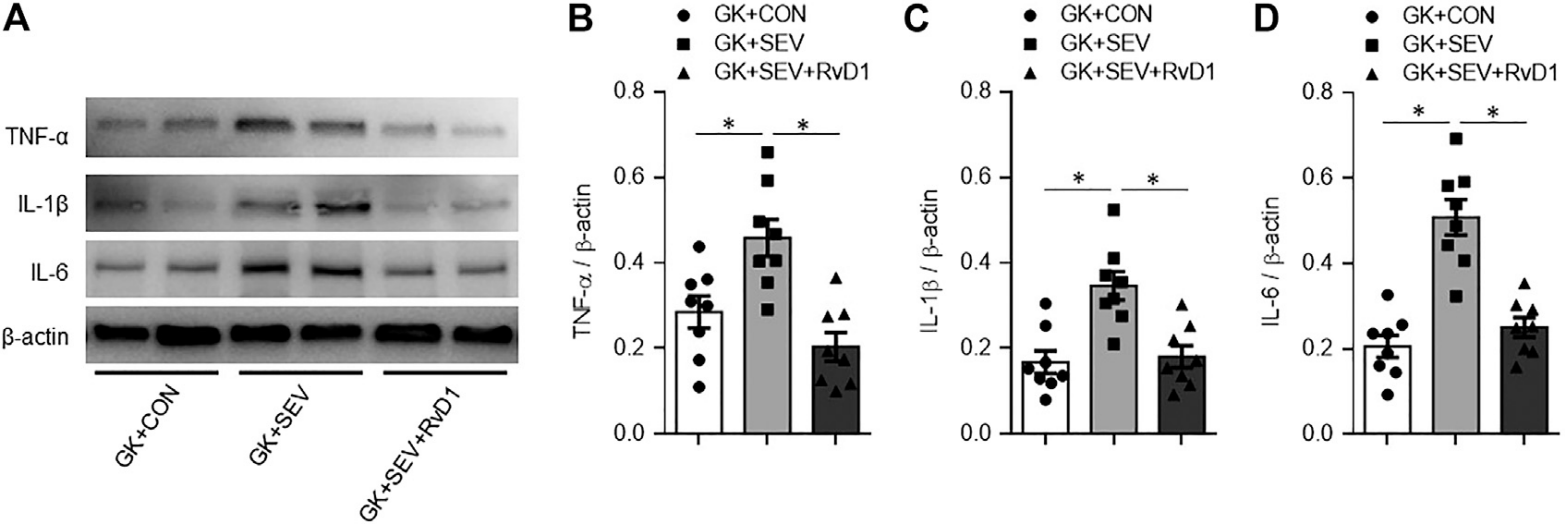
Representative Western blots **(A)** and quantitative comparison of protein expression for pro-inflammatory cytokines TNF-α **(B)**, IL-1β **(C)** and IL-6 **(D)** in the hippocampus in type 2 diabetic Goto-Kakizaki (GK) rats after control (CON) or sevoflurane (SEV) exposure and in exogenous RvD1-treated GK rats after SEV exposure. Data are presented as mean ± SE (n = 8 for each group). *p < 0.05.

Compared with GK + CON rats, GK + SEV rats exhibited significant increase in protein expression of proinflammatory cytokines TNF-α (Figures 3A,B), IL-1β (Figures 3A,C) and IL-6 (Figures 3A,D) in the hippocampus. SEV-induced increase in expression of these proinflammatory cytokines in the hippocampus of GK rats were reduced by treatment with exogenous RvD1.

### Effects of Exogenous Resolvin D1 Treatment on Sevoflurane-Induced Microglia Activity in Diabetic Goto-Kakizaki Rats

**FIGURE 4:**
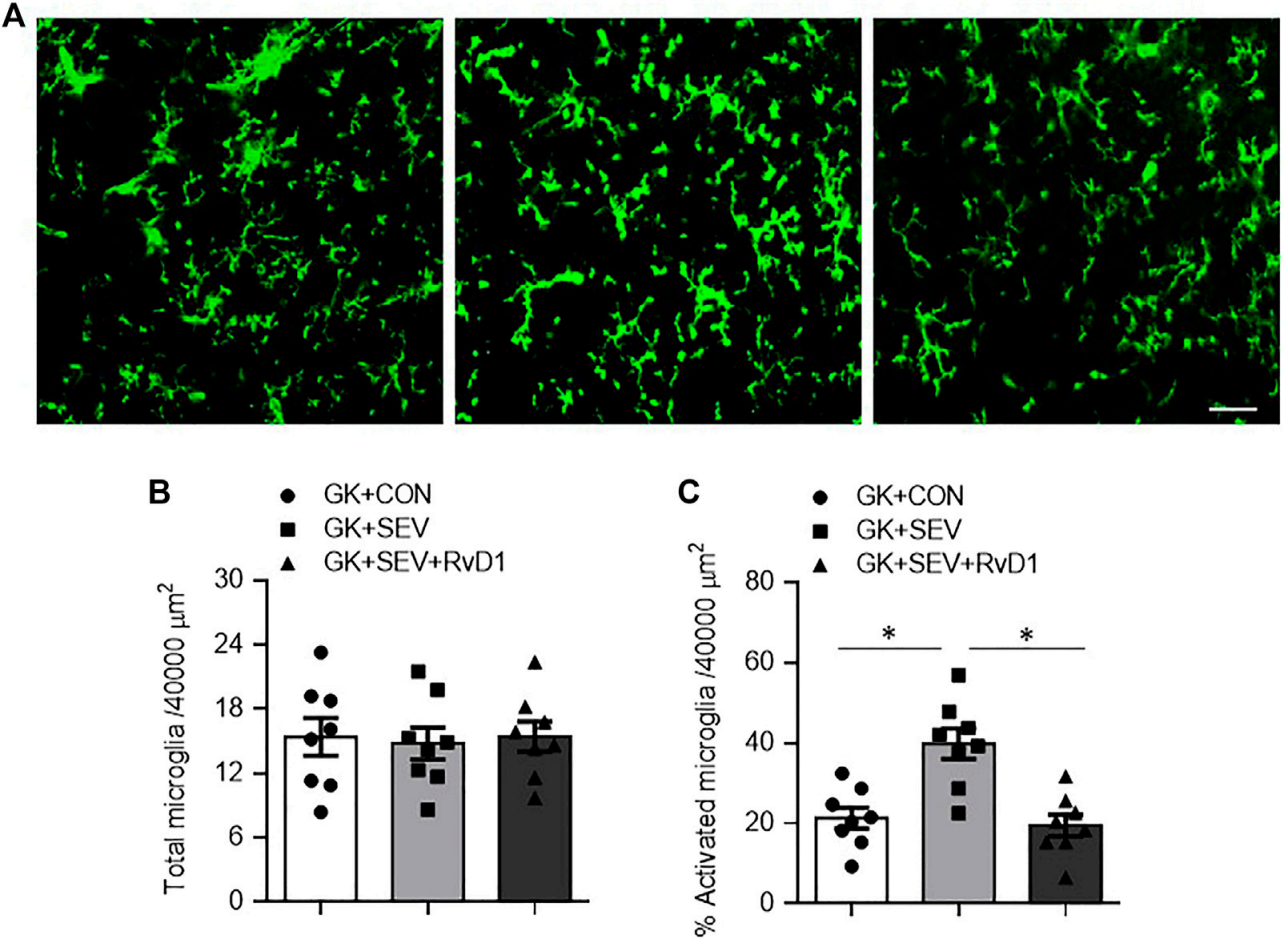
Representative photomicrographs showing CD11b-immunoreactive microglia **(A)** and quantitative comparison of total **(B)** and activated **(C)** microglia in the in the hippocampus in type 2 diabetic Goto-Kakizaki (GK) rats after control (CON) or sevoflurane (SEV) exposure and in exogenous RvD1-treated GK rats after SEV exposure. Scale bar: 20 μm. Data are presented as mean ± SE (n = 8 for each group). *p < 0.05.

Neuroinflammation is initiated by microglia, which are the primary resident immune cells of the central nervous system (CNS). Microglia become activated following exposure to a variety of stimuli, including anesthetics (Rana et al., 2010; Bitzer-Quintero and Gonzalez-Burgos, 2012; Ye et al., 2013). We therefore examined the effect of RvD1 treatment on microglia activation using immunofluorescence study. As shown in Figure 4, there was no difference in the number of total microglia in the hippocampus across three groups (Figures 4A,B). However, the number of activated microglia that were defined by strong CD11b immunoreactivity, an enlarged soma, fewer and shorter processes, was significantly increased in the hippocampus in GK + SEV rats as compared to GK + CON rats (Figures 4A,C). RvD1 treatment of GK + SEV rats did not alter the number of total microglia, but it attenuated the number of activated microglia in the hippocampus induced by SEV exposure.

### Effects of Exogenous Resolvin D1 Treatment on Sevoflurane-Induced Cognitive Decline in Diabetic Goto-Kakizaki Rats

**FIGURE 5:**
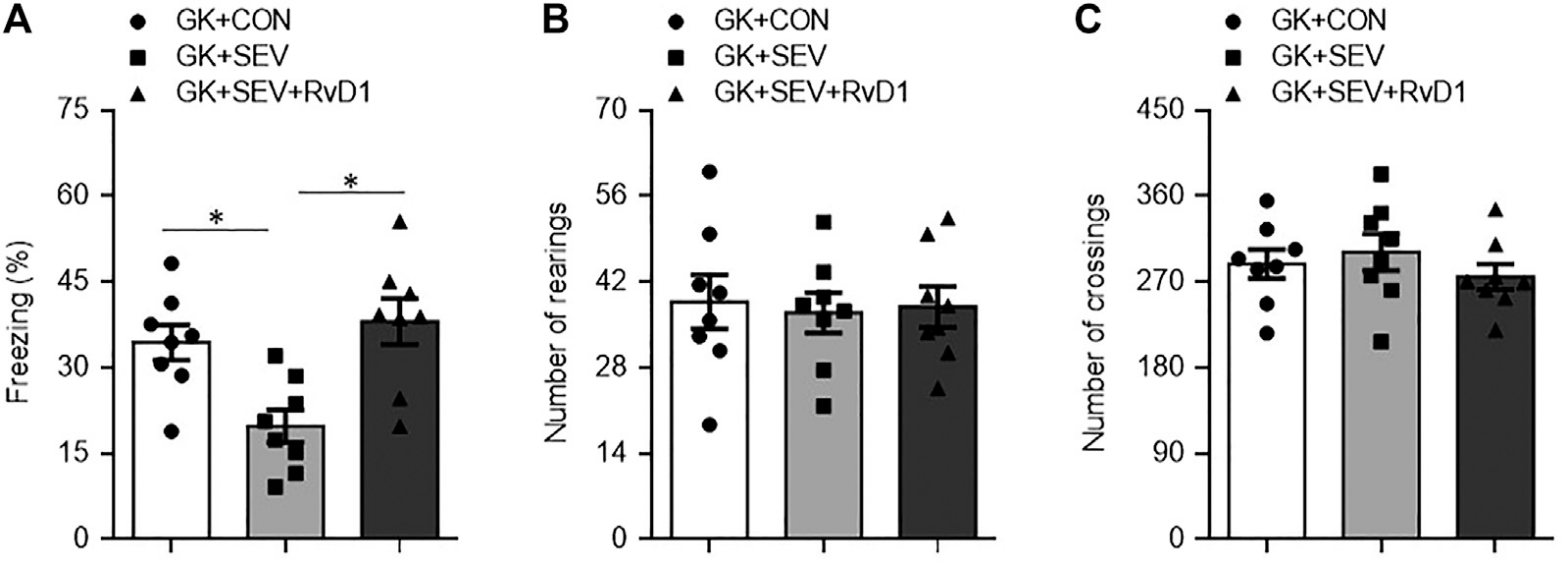
Freezing times in fear conditioning task **(A)** and number of rearings **(B)** and crossings **(C)** in open-field test in type 2 diabetic Goto-Kakizaki (GK) rats after control (CON) or sevoflurane (SEV) exposure and in exogenous RvD1-treated GK rats after SEV exposure. Data are presented as mean ± SE (n = 8 for each group). *p < 0.05.

Neuroinflammation in the hippocampus has been shown to play a key role in the initiation and development of cognitive dysfunction. We further examined the effect of RvD1 treatment on hippocampal-dependent memory with TFC. Compared with GK + CON rats, GK + SEV rats exhibited significantly less freezing 1 week after SEV exposure (Figure 5A), indicating that SEV exposure induces impairment in hippocampal-dependent memory at this time point. Importantly, SEV exposure-induced decrease in freezing in GK rats was significantly increased by RvD1 treatment.

To exclude the difference in general locomotor activity, a potential confounding factor that might influence the assessment of cognitive function, we performed open-field test to assess spontaneous activity following TFC. No significant difference was found between groups for the number of rearings (Figure 5B) and the number of crossings (Figure 5C).

## Discussion

The novel findings of the present study are: 1) Type 2 diabetic GK rats, compared with Wistar rats, have lower levels of RvD1 in the periphery and CNS, which are accompanied by decreased expression of its receptor FPR2 in the hippocampus; 2) SEV exposure elevates levels of RvD1 in the periphery and CNS and increases expression of FPR2 in the hippocampus in Wistar rats but not in diabetic GK rats; 3) SEV exposure-induced neuroinflammation in the hippocampus and cognitive decline in diabetic GK rats are prevented by exogenous RvD1, which increases levels of RvD1 in the periphery and CNS and upregulates expression of FPR2 in the hippocampus. Collectively, these results indicate defects in RvD1 proresolution pathway in the CNS in type 2 DM, which may enhance the sensitivity and susceptibility to the insult from SEV exposure, causing exaggerated neuroinflammation and cognitive decline. Restoration of RvD1 proresolution pathway with exogenous RvD1 can attenuate SEV exposure-induced neuroinflammation in the hippocampus and prevent cognitive decline under type 2 DM conditions.

Inflammatory processes within the CNS are responsible for the development of neurodegenerative and psychiatric diseases including POCD. These processes are associated with the augmented and disturbed activation of microglia and the elevated production of proinflammatory mediators (Trojan et al., 2020). Recent evidence shows that the disruption of the process of resolution of inflammation may be the cause of CNS disorders (Trojan et al., 2020). The resolution of inflammation is regulated by SPMs including RvDs, which interact with specific membrane receptors (Trojan et al., 2020). SPMs are biosynthesized in response to inflammatory challenges to exert potent pro-resolving and anti-inflammatory actions as well as neuroprotective properties (Hansen et al., 2018; Trojan et al., 2020). RvDs synthesis occurs *via* formation of 17-hydroxydocosahexaenoic acid (17-HDHA) from docosahexaenoic acid (DHA) by the action of lipoxygenases (LOX)-15. The 17-HDHA is then converted by 5-LOX into RvD1 and RvD2, which act through the binding to their receptors GRP 32 and FPR2 (Serhan, 2017). FPR2 is widely distributed in the brain including the hippocampus and especially functionally expressed in microglia (Rey et al., 2016; Ho et al., 2018). A previous study reported that levels of 15-LOX and 5-LOX, two enzymes for RvDs synthesis, and expression of FPR2 were increased in microglial cells after LPS application (Rey et al., 2016). In the present study, we found that diabetic GK rats, compared with Wistar rats, exhibited lower levels of RvD1 but not RvD2 in the plasma and CSF, along with decreased expression of FPR2 in the hippocampus. Moreover, SEV exposure failed to increase the levels of RvD1 in plasma and CSF as well as expression of FPR2 in the hippocampus in diabetic GK rats. Our previous study has shown that SEV exposure induces microglia activity and neuroinflammation in the hippocampus and cognitive decline in diabetic GK rats but not in Wistar rats (Li et al., 2017). Taken together, these observations suggest that RvD1-mediated proresolving and anti-inflammatory signaling pathway in the brain is defective in type 2 DM, which might enhance the vulnerability of the brain to inflammatory insults including SEV exposure, causing activation of microglia and neuroinflammation in the hippocampus and subsequent cognitive decline. Our findings are consistent with a previous study showing that type 2 DM impaired RvD1-mediated resolution of inflammation in peripheral tissues as evidenced by decreased levels of 17-HDHA (a marker of RvDs biosynthesis), leading to delayed healing of diabetic wounds (Tang et al., 2013). We speculate that decreased levels of 17-HDHA in type 2 DM might account for lower levels of RvD1 in the plasma and CSF as well as decreased expression of FPR2 in the hippocampus in type 2 diabetic GK rats observed in our study.

Recent studies reported that levels of endogenous RvD1 but not RvD2 in the plasma and brain were decreased in a rat model of parkinson’s disease (Krashia et al., 2019) or rats after focal brain damage (Bisicchia et al., 2018), which were associated with increased microglia activity and neuroinflammation in the brain. Restoration of RvD1 levels with exogenous RvD1 attenuated microglia activity and neuroinflammation, improving neuronal dysfunction and motor deficits in these animal models (Bisicchia et al., 2018; Krashia et al., 2019). Exogenous RvD1 treatment also attenuated microglia activity and neuroinflammation, reduced infarct area and alleviated neurological deficits in rats after neonatal hypoxic-ischemic injury (Liu et al., 2019). *In vitro* study demonstrated that RvD1 treatment decreased LPS-induced proinflammatory cytokine expression in microglial cells (Rey et al., 2016). We therefore examined whether treatment with exogenous RvD1 would prevent microglia-mediated neuroinflammation and cognitive decline in diabetic GK rats after SEV exposure. Consistent with previous results (Li et al., 2017), SEV exposure caused significant increases in microglia activity and proinflammatory cytokine expression in the hippocampus in the diabetic GK rats, which were associated with cognitive decline as indicated by less freezing in the TFC test. Systemic RvD1 administration robustly elevated levels of RvD1 in both plasma and CSF in diabetic GK rats, suggesting that RvD1 is capable of crossing the blood-brain barrier under diabetic conditions. Moreover importantly, we found that RvD1 treatment of diabetic GK rats attenuated SEV exposure-induced increases in microglia activity and proinflammatory cytokine expression in the hippocampus, preventing SEV exposure-induced cognitive decline. Notably, the number of line crossing and the frequency of rearing performed in open-field test were comparable among the three diabetic groups, which excluded the possibility that the behavioral assessment was affected by altered spontaneous movement. Additionally, there were no differences in metabolic parameters across the three diabetic groups, suggesting that benefic effects of RvD1 treatment in SEV exposure-induced neuroinflammation and cognitive dysfunction in diabetic GK rats could not be attributed to improvements in metabolism.

Several limitations of the present study deserve consideration. First, effect of RvD1 treatment on astrogliosis was not examined. It is now clear that astrocytes, the most abundant glial cells in the CNS, also play a critical role in regulation of neuroinflammation (Giovannoni and Quintana, 2020; Tiberi and Chiurchiu, 2021). Activated astrocytes in response to many CNS insults lead to astrogliosis, which is a hallmark of neuroinflammation and characterized by a higher production of proinflammatory cytokines and reactive species and a lower production of neurotrophic factors (Tiberi and Chiurchiu, 2021). Astrocytes have been shown to express high levels of FPR2 (Tiberi and Chiurchiu, 2021). A previous study reported that RvD1, which acts through the binding to FPR2, attenuates both microglial and astrocyte activation and reduces neuroinflammation in the brain of rats after focal brain damage (Bisicchia et al., 2018). Thus, we could not exclude the possibility that RvD1 treatment reduced SEV-induced neuroinflammation in diabetic GK rats in part by attenuating astrogliosis. Second, the FPR2 expression in the hippocampus was assessed by Western blot, which could not determine the alteration of FPR2 expression in a specific cell type. Further studies are needed to determine FPR2 expression on microglia in the different experimental conditions using confocal immunofluorescence study. Finally, the levels of RvD1 and RvD2 were measured with commercial ELISA kits instead of a precise technique. Further studies are necessary to confirm the levels of RvD1 and RvD2 by more precise techniques such as the gold standard liquid chromatography mass spectrometry.

In summary, the present study demonstrates that RvD1 proresolution pathway is impaired in the CNS in diabetic GK rats. This lack of anti-inflammatory and proresolving lipid mediators may enhance the sensitivity and susceptibility to SEV exposure, which leads to exaggerated microglia activity and neuroinflammation in the hippocampus, contributing to cognitive decline under type 2 DM conditions. Restoration of RvD1 proresolution pathway in diabetic GK rats with exogenous RvD1 can prevent SEV exposure-induced cognitive decline by attenuating microglia-mediated neuroinflammation in the hippocampus. Targeting RvD1 proresolution pathway in the CNS may provide a novel option for prevention of anesthetic SEV-induced cognitive decline in patients with type 2 DM.

## Data Availability

The original contributions presented in the study are included in the article/supplementary material, further inquiries can be directed to the corresponding authors.
